# A clinical study on safety and efficacy of Naesohwajung-tang on functional dyspepsia

**DOI:** 10.1097/MD.0000000000019910

**Published:** 2020-05-22

**Authors:** Na-Yeon Ha, Seulki Kim, Seok-Jae Ko, Jae-Woo Park, Jinsung Kim

**Affiliations:** a Department of Gastroenterology, College of Korean Medicine, Kyung Hee University, Seoul, Republic of Korea; b Department of Clinical Korean Medicine, Graduate School, Kyung Hee University, Seoul, Republic of Korea.

**Keywords:** functional dyspepsia, herbal medicine, *Naesohwajung-tang*, randomized controlled trial

## Abstract

**Background:**

Functional dyspepsia (FD) is a chronic gastrointestinal disorder diagnosed with dyspeptic symptoms, such as recurrent or persistent postprandial fullness, epigastric pain, burning, and early satiety without any evidence of structural abnormality. In traditional Korean medicine, herbal remedies are one of the preferred treatments for gastrointestinal symptoms. Among them, *Naesohwajung-tang* (NHT) has been frequently prescribed to improve dyspeptic symptoms for a long time. However, the safety and efficacy of NHT still remain unclear. Therefore, this study aims to evaluate the safety and efficacy of NHT in patients with FD using a randomized controlled trial.

**Methods:**

This is a protocol for a multi-center, randomized, double-blind, placebo-controlled trial. Total 116 participants aged between 19 and 75 years diagnosed with FD according to the Rome IV diagnosis criteria will be recruited at two Korean medicine hospitals. Eligible participants will be randomly assigned to either a NHT group or a placebo group in a 1:1 ratio. Each participant will be instructed to take NHT or placebo granules three times a day for 4 weeks. The primary outcome is the change in the total dyspepsia symptom score to confirm the efficacy of NHT. The secondary outcomes include overall total effect, single dyspepsia symptom scale, food retention questionnaire, *Damum* questionnaire, FD-related quality of life, electrogastrography, and safety assessments. Adverse events will be evaluated at every visit. The primary endpoint will be measured at week 4, and then re-evaluated at week 8 through a follow-up phone visit.

**Discussion:**

This trial will evaluate the safety and efficacy of NHT as a treatment of FD. The results of this trial will not only verify whether NHT improves FD symptoms and gastric myoelectrical activity, but also clarify those correlations.

**Trial registration number:**

CRIS KCT0003405.

## Abstract 2

Supplemental Digital Content is available in the text

## 1 Introduction

Functional dyspepsia (FD) is a functional gastrointestinal disorder that is characterized by one or more of the following symptoms; bothersome postprandial fullness, early satiation, epigastric pain, and epigastric burning that cannot be explained by any organic disease.^[1]^ The prevalence of FD ranges from 8% to 23% in Asia and from 10.3% to 20.4% in South Korea.^[2–4]^ In addition to the gastric symptoms, people with FD also suffer from abnormal daily activities and poor quality of life due to direct or indirect economic burden.^[5]^ Although the pathology of FD is unclear, there are several hypotheses that explain the mechanisms of disease. Specifically, abnormal gastric motility has been observed in FD patients as the previous studies.^[6,7]^

The Rome IV diagnostic criteria divide FD into two different subcategories:

1. postprandial distress syndrome (PDS), which is characterized by bothersome postprandial and/or bothersome early satiation, and
2. epigastric pain syndrome (EPS), which is characterized by bothersome epigastric pain and/or bothersome epigastric burning.^[8]^

PDS is more prevalent than EPS in Asia,^[4,9]^ and is associated with abnormal gastric motility and gastric emptying.^[10]^

Patients with FD are conventionally treated with proton pump inhibitors, histamine-2 receptor antagonists, antidepressants, prokinetic agents, or antibiotics to eliminate *Helicobacter pylori* infection.^[11–14]^ Because of the limitations and complications of these medications, many FD patients tend to prefer alternative treatment options, such as herbal medicine.^[15–17]^*Naesohwajung-tang* (NHT) is one of the most frequently prescribed herbal medicine for treating FD in traditional Korean medicine. Although some studies have showed that NHT is effective in the treatment of FD,^[18,19]^ randomized controlled studies to provide clinical evidence of NHT as a treatment option for patients with FD have not yet been performed. The goal of this study is to propose a protocol of randomized, placebo-controlled trial aimed to evaluate the safety and efficacy of NHT in patients with FD.

## 2 Methods and design

### 2.1 Objective

This study aims to assess the safety and efficacy of NHT in patients with FD.

### 2.2 Hypothesis

We hypothesize that the administration of NHT for 4 weeks will relieve the symptoms of FD.

### 2.3 Design

**Figure 1:**
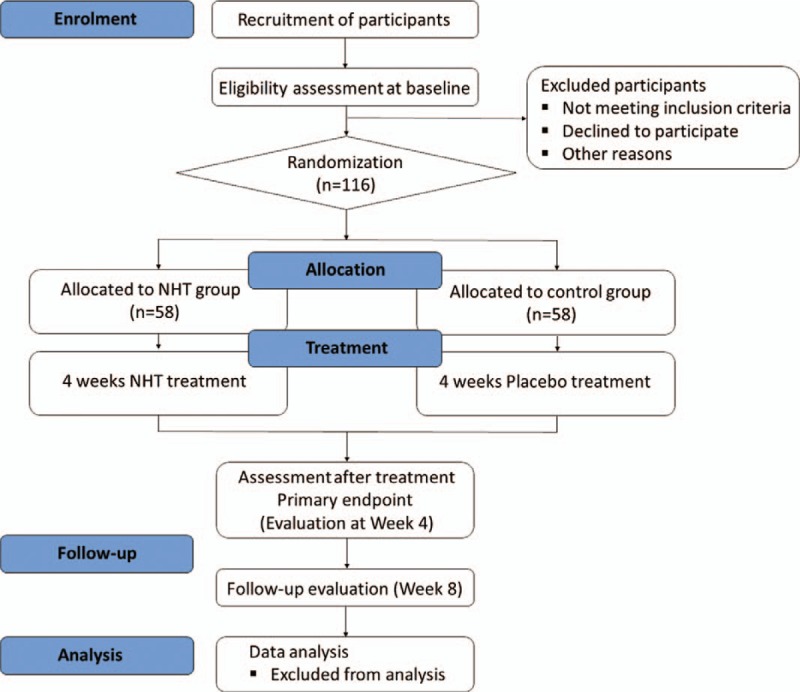
Flow chart of the trial process. NHT = *Naesohwajung-tang*.

**Figure 2:**
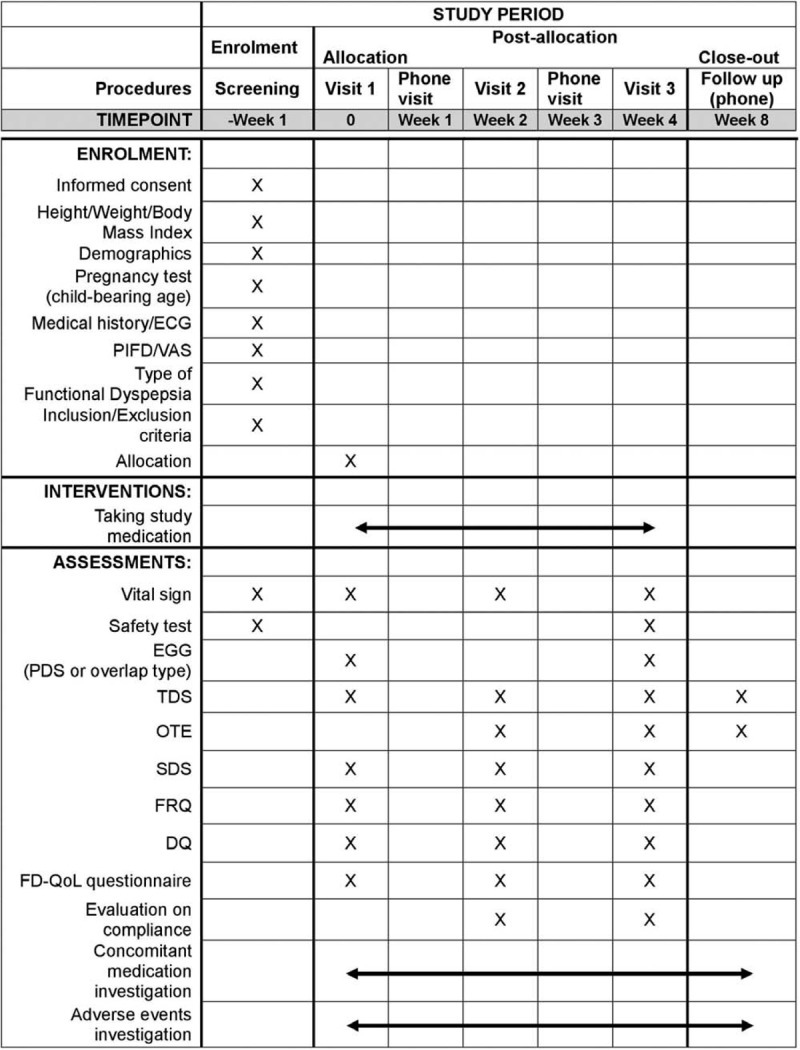
SPIRIT figure showing the schedule of enrollment, interventions, and assessments. DQ = *Damum* questionnaire, ECG = electrocardiography, EGG = electrogastrography, FD-QoL = functional dyspepsia-related quality of life, FRQ = food retention questionnaire, OTE = overall total effect, PIFD = pattern identification of functional dyspepsia, SDS = single dyspepsia symptom, TDS = total dyspepsia symptom, VAS = visual analogue scale.

This study is a prospective, multi-center, randomized, double-blind, and placebo-controlled trial. It will be conducted at Kyung Hee University Korean Medicine Hospital and Kyung Hee University Hospital at Gangdong, Seoul, Korea, from May 2019 to December 2020. This trial has been registered with the Clinical Research Information Service (CRIS) (KCT0003405, registered on December 24, 2018). Total 116 participants who meet the study criteria and consent to participate in trial will be randomly assigned into either NHT or placebo group with a ratio of 1:1. They will undergo 4 weeks of administration and another 4 weeks of follow-up period. The study flow chart illustrating the entire study procedure is shown in Figure 1 and Figure 2 shows the schedule of outcome measurements, following the Standard Protocol Items: Recommendations for Interventional Trials (SPIRIT) checklist (see Additional file 1).

### 2.4 Ethics approval

The Research Ethics Committees at Kyung Hee University Korean Medicine Hospital (approval number is KOMCIRB-2017–08-030) and Kyung Hee University Hospital at Gangdong (No. KHNMCOH 2019-01-003-003) have approved this protocol. Before enrollment, all participants will sign a written informed consent form after fully informed of the purpose, process and risks associated with the trial by the investigators. The protocol is in accordance with the revised version of the Declaration of Helsinki and Good Clinical Practice guidelines approved by the Korea Food and Drug Administration.

### 2.5 Sample size calculation

We hypothesize that oral administration of NHT is more effective than that of placebo in relieving FD symptoms. As the first randomized clinical trial of NHT in patients with FD, we referred to a previous study with a modified design using the same primary outcome.^[20]^ The previous study showed 1.4 points of improvement (μ_c_ – μ_t__=_*δ*) in the TDS scores for FD patients after 4 weeks of treatment with a traditional Chinese herb. In this study, the ratio (λ) of the experimental group to the control group will be 1:1, and the sample size was calculated at the 5% significance level (α) and 80% power (1 – β). The formula used to estimate the sample size is as follows, assuming that the standard deviations (SD = σ) of two groups are different and applying the larger variance in common (
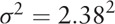
): 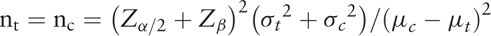


Considering the dropout rate as 20%, we calculated the necessary sample size to be 116 participants (58 in each group).

### 2.6 Participants

#### 2.6.1 Inclusion criteria

We will include the participants who meet the Rome IV criteria of FD in Table 1, classified into PDS and/or EPS. The criteria must be fulfilled for the last 3 months with symptom onset at least 6 months before diagnosis.^[1]^ The inclusion criteria for this trial are shown in Table 2.

**Table 1:**
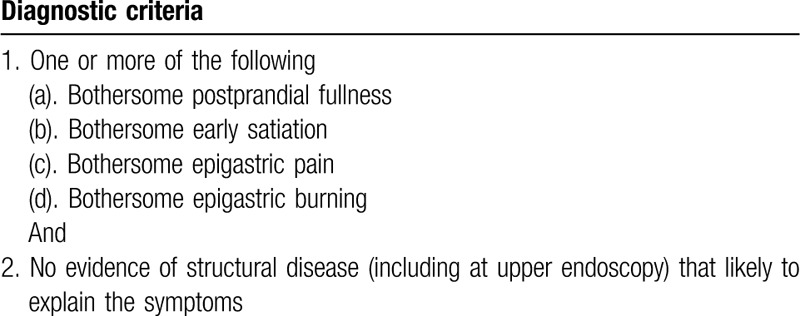
Rome IV criteria of functional dyspepsia.

**Table 2:**
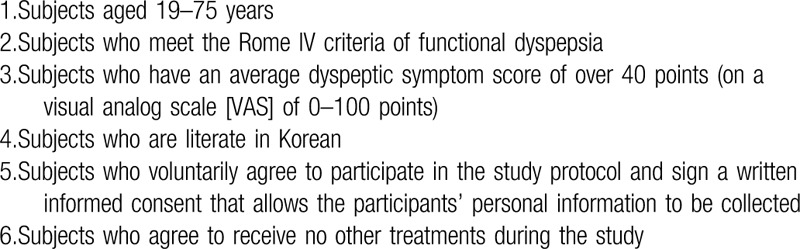
Inclusion criteria in the trial.

#### 2.6.2 Exclusion criteria

Participants will be excluded if they meet the criteria listed in Table 3.

**Table 3:**
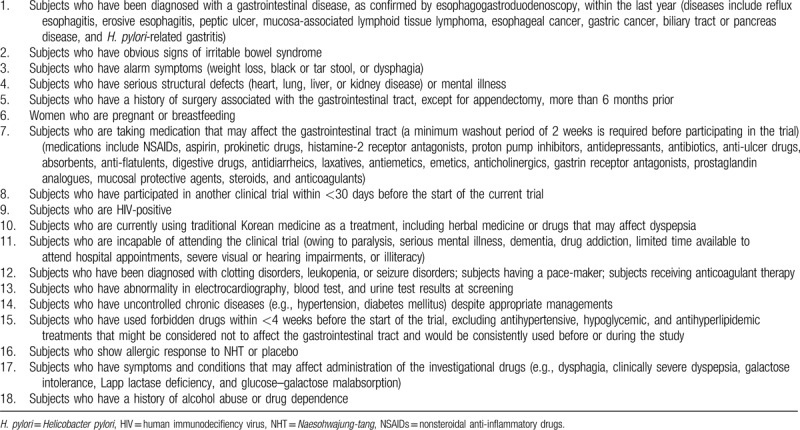
Exclusion criteria in the trial.

### 2.7 Recruitment

Advertisements will be placed on the notice boards at Kyung Hee University Korean Medicine Hospital and Kyung Hee University Hospital at Gangdong, or posted on the local public transportation.

### 2.8 Randomization and allocation concealment

For randomization, an independent statistician who is not involved in the clinical aspects of the trial will generate 116 random numbers, using a randomization sequence generator the PROC PLAN of SAS 9.4 (SAS Institute Inc, Cary, NC). A manufacturer will package the investigational drugs identically to conceal the intervention allocation and label with numbers from 1 to 58 for each center (58 in each group and distributed equally to both centers).

Eligible 116 participants will be assigned into either NHT or placebo group with a consecutive random number, according to predetermined allocation list. The statistician will keep random number tables concealed within sealed opaque envelopes until the end of the study.

### 2.9 Blinding

Participants, investigators, clinical research coordinators (CRCs), and pharmacists in each hospital will be blinded to group allocation throughout the study period. The one who will be associated with the randomization is only the statistician. The NHT and placebo granules are indistinguishable in appearance from each other by the naked eye, smell, or taste. The investigational drugs will be delivered to each hospital in a concealed manner to maintain blinding. The CRCs will enroll participants and assign randomization numbers sequentially at each center. The pharmacists will distribute the packaged NHT or placebo to the subjects according to random number.

### 2.10 Unblinding in an emergency

If the investigators find any serious adverse events or suspect any unexpected serious adverse reactions, the event must be assessed and immediately reported to the principal investigator and the Institutional Review Board (IRB). The principal investigator will decide whether blinding should be revealed. The investigator must inform the statistician of participant's randomization number, and then the statistician must verify whether the drug taken by subject is a real NHT or placebo. In addition, these events will be recorded in the case report file (CRF).

### 2.11 Withdrawal criteria

Participants are allowed to withdraw from the trial at any time during the study period. The investigators will identify and record the reason of withdrawal. The criteria for dropping out of the study are below:

- when surgery or hospitalization treatment is necessary to participant due to accidents or other diseases.
- when participant requests to stop the study or withdraw consent to participate.
- when the principal investigator judges that an unavoidable reason to terminate the study has occurred.
- in case of serious violation of the study protocol by investigator or participant.
- in case of receiving a combination treatment with prohibited drugs or therapies related to FD during the study period.

## 3 Intervention

### 3.1 Trial drugs

#### 3.1.1 NHT

NHT has been frequently used as herbal medicine to alleviate dyspeptic symptoms for a long time. It is a combined prescription of *Daehwajung-eum* and *Naeso-san,* which were both described as therapies for dyspepsia in *Bangyakhappyeon*, a representative textbook of traditional Korean medicine.^[21]^ For this study, brown and bitter granules of NHT herbal extract are produced at Shinhwa Pharmaceutical Corp. (Daegu, Republic of Korea) in compliance with the Korean Good Manufacturing Practice (KGMP) guidelines.

Approximately 5.0 g of solid extract can be produced after boiling and filtering 26.73 g of herb mixture. After mixing cornstarch (0.6 g), powdered cellulose (1.4 g), and moderate amounts of purified water to the extract and then dried in granule form, we can obtain NHT granules (7.0 g), consisting of the following 19 herbs in Table 4. The NHT group will take NHT granules three times a day at 30 min after each meal for 4 weeks.

**Table 4:**
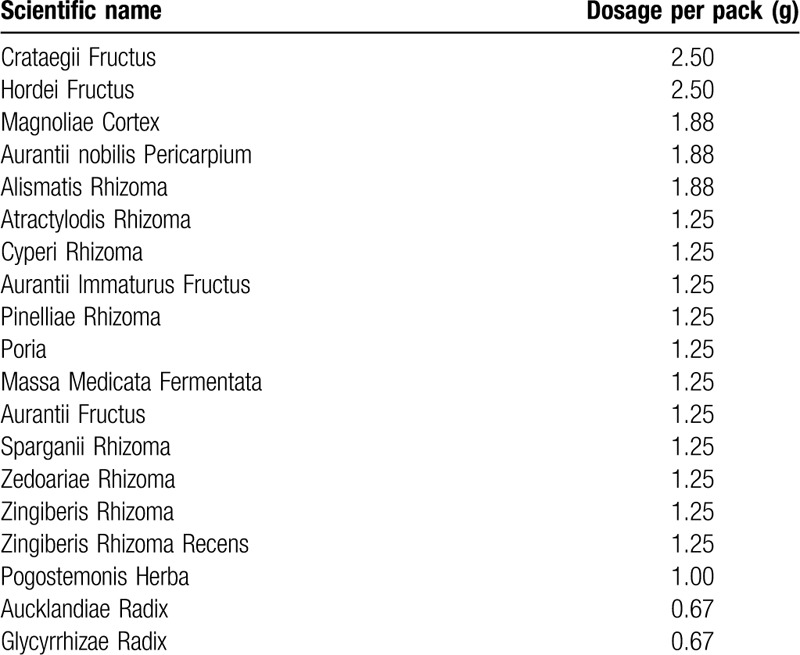
Herbal plants contained in *Naesohwajung-tang.*

#### 3.1.2 Placebo

The placebo granules are produced in the same facility as NHT granules, using the standard methods of placebo manufacturing based on the KGMP guidelines. The placebo granules are indistinguishable from the real NHT granules in taste, shape, weight, color, and flavor so that participants will not recognize whether they are administered for the treatment group or the control. However, the placebo does not have any components effective for treating FD. The placebo granules (7.0 g) consists of the following components; lactose hydrate (3.268 g), cornstarch (3.080 g), AF cacao color 280 (0.249 g), Sepia color powder (0.300 g), Food Red No. 40 (0.005 g), *Ssangwha* herbal flavor (0.098 g), and moderate amounts of purified water. In addition, the placebo is packaged into small sachets and labeled same as the real NHT with opaque aluminum bags. The control group will receive placebo three times a day at 30 min after each meal for 4 weeks.

Participants of each group will receive the investigational drugs at visit 1 (week 0) and visit 2 (week 2). In order to evaluate the suitability of packaging and storage conditions, and the physicochemical and biological stability of drugs during the study period, several quality assurance tests and stability tests including heavy metal analysis and microbial testing will be conducted.

### 3.2 Concomitant and forbidden drugs

Participants are not allowed to take drugs that may affect gastrointestinal tract during the study. If necessary, a wash-out period of at least 2 weeks is required before beginning the study. Any participant having received a treatment of Korean medicine to alleviate FD symptoms will be prohibited from participation. However, taking medication for the management of underlying chronic diseases, such as hypertension, diabetes mellitus, and hyperlipidemia, will be allowed. Participants will be instructed to report all the concomitant drugs and treatments conducted before, on every visit.

### 3.3 Outcome measures

#### 3.3.1 Primary outcome

The total dyspepsia symptoms (TDS) scale is the primary outcome measurement to assess the change of dyspeptic symptoms. If the TDS scores show significant differences between two groups after the administration of NHT (week 4), NHT will be regarded as an effective treatment of FD. The TDS scale is comprised of eight items; postprandial fullness and bloating, early satiety, epigastric pain, epigastric burning, nausea, vomiting, belching, and other symptoms. Each item is based on a 4-point Likert scale, and higher scores are positively correlated with more severe dyspeptic discomforts.^[22]^ TDS will be evaluated at baseline, week 2, week 4, and week 8.

#### 3.3.2 Secondary outcomes

1. Overall treatment effect (OTE): To assess OTE, participants will be asked the following question: “How do you feel about the overall treatment effect throughout the past treatment period with respect to your quality of life and dyspeptic symptoms?” The participants will answer based on a 15-point Likert scale ranging from extreme aggravation (−7) to complete relief (+7).^[23]^ OTE will be assessed at week 2, week 4, and week 8.
2. Single dyspepsia symptom (SDS) scale: To subordinately assess the severity of dyspeptic symptoms, four aspects of the typical symptoms of FD will be measured based on a 4-point Likert scale; epigastric pain, epigastric burning, postprandial fullness and bloating, and early satiety. Each symptom will be scored in terms of frequency, intensity, and a level of discomfort.^[22]^ SDS will be evaluated at baseline, week 2, and week 4.
3. Food retention questionnaire (FRQ): In traditional Korean medicine, food retention is a pathological process that results in symptoms such as abdominal fullness or pain, indigestion, and water brash.^[24]^ FRQ consists of 17 items based on a 7-point Likert scale ranging from disagree very strongly to agree very strongly.^[25]^ The Likert scores of 1, 2, 3, and 4 will be transformed to 0 point, and 5, 6, and 7 to 1 point. If the total score is 6 or more, we will describe the participant as having food retention. FRQ will be measured at baseline, week 2, and week 4.
4. *Damum* questionnaire (DQ): DQ is a useful tool for diagnosing a phlegm pattern in traditional Korean medicine, characterized by symptoms such as indigestion, poor appetite, fatigue, and dizziness.^[26]^ DQ consists of 14 items with a 7-point Likert scale ranging from disagree very strongly to agree very strongly. Higher scores suggest higher likeliness of having phlegm.^[27]^ DQ will be measured at baseline, week 2, and week 4.
5. FD-related quality of life (FD-QoL) questionnaire: The FD-QoL questionnaire comprises 21 items from four categories; diet, daily activity, emotion, and social functioning.^[28]^ Each item will be evaluated based on a 5-point Likert scale, with higher scores indicating worse quality of life. FD-QoL will be evaluated at baseline, week 2, and week 4. The investigators will be trained thoroughly to ensure all the questionnaires completed accurately and unbiasedly.
6. Gastric myoelectrical activity (GMA): GMA will be measured by using a surface multichannel electrogastrography (EGG) (Polygraf ID, Medtronic A/S, Denmark) at baseline and week 4 to detect abnormal gastric motility in PDS or overlap group. The measurement will be performed as described in the previous studies.^[29,30]^ The multichannel EGG consists of three identical amplifiers with a recording range of 1.0 to 12.0 cycles/min. To measure GMA, three pre-gelled active surface electrodes will be applied to pre-abraded skin, corresponding to the corpus (channel 1), the proximal antrum (channel 2), and the distal antrum (channel 3). A reference electrode and a ground electrode will be also attached to abdominal skin. All recordings will be performed in a quiet room and participant will abstain from food intake overnight for at least 6 h. The subject will be kept in a supine position and instructed not to talk, move, or sleep during assessment to avoid interferences that may affect the results. EGG will be performed in three steps. First, a preprandial recording (30 min) will be conducted during the fasting state. Second, the subject will consume 400 mL of a nourishing beverage (New Care; Wellife, Seoul, Republic of Korea). Finally, a postprandial recording (30 min) will be conducted. The EGG dominant frequency and power, percentage of normal/brady/tachygastria and gastric arrhythmia, percentage distribution of EGG power, and post-preprandial power ratio will be analyzed to assess gastric motility. The investigators who operate EGG will receive training before starting the trial to enhance the accuracy of a measurement.
7. Standard tool for pattern identification of functional dyspepsia (PIFD): The standard tool for PIFD has been developed after searching for the literature such as Korean and Chinese clinical trials and research articles to confirm the patterns of dyspeptic symptoms. Next, the expert committee assessed the validity of translations and discussed clinical applications of the questionnaire through the Delphi method. This standard tool consists of 40 items including tongue and pulse diagnosis, classifying FD patients into six patterns of identification; pattern/syndrome of
  (a) deficiency (with cold) of the spleen system,
  (b) spleen deficiency with *qi* stagnation,
  (c) disharmony of the liver and stomach systems,
  (d) tangled cold and heat,
  (e) dampness-heat encumbering the spleen system, and
  (f) food retention.

Each weighted value reflects the importance of each symptom. PIFD will be evaluated at screening, and each pattern will be compared with other outcomes.

### 3.4 Safety assessment

The following clinical laboratory parameters will be examined to assess the safety of NHT treatment at screening and week 4; white blood cell, red blood cell, hemoglobin, hematocrit, mean corpuscular volume, mean corpuscular hemoglobin, mean corpuscular hemoglobin concentration, mean platelet volume, platelet, aspartate aminotransferase, alanine aminotransferase, gamma-glutamyl transpeptidase, blood urea nitrogen, creatinine, total cholesterol, triglyceride, high density lipoprotein cholesterol, low density lipoprotein cholesterol, glucose, erythrocyte sedimentation rate, and urinalysis. At screening, these laboratory tests and electrocardiography will be executed to decide whether a subject is eligible to enroll before randomization. In addition, adverse events reporting during the study period will be analyzed to verify the safety of NHT in patients with FD.

### 3.5 Evaluation of compliance

Treatment compliance will be evaluated every 2 weeks during the period of administration by counting the number of returned containers of NHT or placebo granules. Participants with more than 70% compliance will be included in the per-protocol analysis. All researchers will try to increase the compliance of medications, reminding participants of the study schedule and instructing them to keep taking drugs through frequent phone calls.

### 3.6 Adverse events reporting

An adverse event is any unfavorable medical incident experienced by a participant that does not necessarily have a causal correlation with the intervention. All adverse events will be assessed and documented in CRFs at each visit during the entire study period. Proper medical support will be provided to the participant when any adverse event occurs.

A serious adverse event is an adverse event that may result in death, illness requiring hospital admission, life-threatening incident, persistent disability/incapacity, or congenital anomaly/birth defect. The principal investigator will immediately report such cases to the IRB, evaluate and record the time, progress and severity of the events, providing appropriate treatments to minimize and alleviate symptoms. The participants directly affected by the trial will be compensated based on the compensation protocol after consulting the insurance institution.

### 3.7 Data management

For the accurate data collection, highly trained investigators and qualified CRCs will use CRFs, which include only random numbers and no subject identifiers to maintain security. The electronic data will be administered in the Mytrial system (http://www.mytrial.co.kr/) at Bethesdasoft Co., Seoul, Korea. User IDs and passwords that allow access to the database will be provided to only authorized personnel. The data management system will be designed to allow electronic data recorded and audited by the automatic editing system (e.g., proofreading of data according to the reference values). The completed CRFs will be locked not to be changed and the data will be analyzed by an independent statistician.

### 3.8 Quality control

To maintain accuracy and quality of the study procedure, data monitoring will be conducted by the Unique Marketing & Training Co., a contract research organization located in Seoul, Korea. The authorized clinical research associate (CRA) will confirm the enrollment, blinding, and withdrawal procedures, and CRF completion thoroughly using the standard operating procedures. All the study files, such as informed consent forms and recorded data in CRFs, will regularly reviewed by the monitoring committee. The data monitoring committee has no competing interests.

### 3.9 Statistical analysis

The efficacy of NHT will be analyzed on the full analysis set and per-protocol analysis. The last observation carried forward method will replace missing data to assess the robustness in the results. The safety of NHT will be assessed for the participants who are randomly assigned and received the investigational drugs at least once. The outcomes will be handled by an independent statistician.

Statistical analysis will be performed by using the SPSS program, version 20.0 (IBM SPSS Statistics, New York). The data will be expressed as mean ± standard deviation or as numbers with proportions. The changes after taking the investigational drugs will be compared between the experimental group and the control group. A chi-square test or Fisher's exact test will be used for discrete variables, while independent two sample *t* test for continuous variables. Those that do not satisfy normality will be analyzed by the Mann–Whitney *U* test as a nonparametric statistical test. The analysis of variance will be used for subgroup analysis of the changes in each pattern identification. The assessment of long-term therapeutic effects in a certain period from allocation to close-out will be performed on the basis of repeated measures design. The statistical relationship between subjective components of each assessment and objective EGG parameters will be analyzed with regression analysis. For safety assessment, the frequency and severity of adverse events and the changes in laboratory measurements will be compared between two groups. *P*-values <.05 will be regarded as statistically significant.

## 4 Discussion

FD is a common disease that results in personal distress and somatic symptoms, and causes high economic burden in the society.^[31,32]^ Currently, conventional drugs available for FD has been prescribed to patients, such as proton pump inhibitors (PPIs), histamine-2 receptor antagonists, and prokinetic agents.^[33]^ However, FD remains a challenging clinical problem because of its heterogeneous characteristics. Because of dissatisfaction with conventional therapies, herbal medicine have attracted FD patients’ attention as an alternative solution.^[34,35]^

Traditional herbal remedies have been generally used for the treatment of FD in traditional Korean medicine. Among them, NHT, a combined prescription of *Daehwajung-eum* and *Naeso-san* consisting of 19 herbs, has been frequently prescribed for dyspepsia for a long time. According to a previous study, it was found that some EGG findings in the postprandial phase were improved after 4-week administration of NHT on FD children with abdominal pain.^[19]^ In animal research on antral dilated rats to evaluate the effect of *Naeso-san*, gastric emptying and myoelectrical activity of gastric smooth muscle were measured. It was confirmed that *Naeso-san* activated gastric motility through the cholinergic pathway, increasing the significant postprandial dominant power and gastric emptying.^[36]^ In addition, *Daehwajung-eum* inhibited contractions on the isolated rat fundus-strip, induced by acetylcholine chloride and barium chloride.^[37]^ Based on these previous studies, NHT is expected to improve dysmotility-related FD symptoms by modulating gastric motility such as gastric adaptive relaxation. However, there is no randomized controlled trial assessing the safety and efficacy of NHT as a treatment of FD. Thus, we aim to obtain objective information about the efficacy of NHT to activate GI motility, and improve both FD symptoms and EGG findings simultaneously.^[38,39]^

EGG is a noninvasive method that uses surface-placed electrodes to evaluate GMA.^[40,41]^ Though there is a heterogeneity of association between dyspeptic symptoms and EGG recordings among previous studies,^[6,42,43]^ EGG is still useful and convenient method to detect abnormal gastric motility. In this study, participants will be subdivided into two subtypes; PDS, which includes overlap type, or EPS. EGG will be used to evaluate gastric motility in PDS group, and the results will be analyzed in comparison to other outcomes.

According to the guidelines for setting endpoint in the clinical trial for FD, it is recommended that 4 weeks of treatment is needed to demonstrate short-term effects of intervention, considering pharmacological effects of drugs and natural history of FD.^[44]^ In addition, 4-week period has been applied in most of clinical studies to investigate curative effects of medicine such as PPIs, prokinetics, and herbal medicine in the treatment of FD.^[33,45]^ Therefore, we set the ideal duration of administration to 4 weeks.

To evaluate the efficacy of NHT, the TDS score will be measured for the primary outcome, whereas OTE, SDS, FRQ, DQ, FD-QoL, and GMA will be studied as secondary outcomes. Comparison of subjective dyspeptic symptoms and objective assessments of gastric activity will improve reliability in research findings. The study will be published regardless of the results, in accordance with the consolidated standards of reporting trials (CONSORT) guidelines.

There are some limitations in this study. First, 4 weeks of follow-up period after treatment ends is relatively short. Second, more frequent implementation of EGG may be helpful for predicting treatment effects. Nevertheless, as the first randomized clinical trial to assess the efficacy and safety of NHT in patients with FD, the results of this study are expected to strengthen the evidence of therapeutic effects of NHT on FD. Thus, it can contribute to the development and implementation of clinical practice guidelines for effective treatment and management of FD, improving public health.

## Acknowledgments

This study was supported by a grant through the project ‘Development of Korean medicine clinical practice guidelines’ of the Guideline Center for Korean Medicine, National Institute for Korean Medicine Development.

## Author contributions

**Conceptualization:** Na-Yeon Ha, Seulki Kim.

**Investigation:** Na-Yeon Ha, Seulki Kim, Seok-Jae Ko.

**Supervision:** Jae-Woo Park, Jinsung Kim.

**Writing – original draft:** Na-Yeon Ha, Seulki Kim.

**Writing – review & editing:** Seok-Jae Ko, Jae-Woo Park, Jinsung Kim.

## Supplementary Material

Supplemental Digital Content

## References

[1] Stanghellini V Chan FK Hasler WL Gastroduodenal disorders. Gastroenterology 2016;150:1380–92. 27147122 10.1053/j.gastro.2016.02.011

[2] Uday CG Rajan S Full-Young C Epidemiology of uninvestigated and functional dyspepsia in Asia: facts and fiction. J Neurogastroenterol Motil 2011;17:235–44. 21860815 10.5056/jnm.2011.17.3.235 PMC3155059

[3] Kim SE Kim NY Lee JY Prevalence and risk factors of functional dyspepsia in health check-up population: a nationwide multicenter prospective study. J Neurogastroenterol Motil 2018;24:603–13. 29938463 10.5056/jnm18068 PMC6175566

[4] Kim SE Park HK Kim N Prevalence and risk factors of functional dyspepsia: a nationwide multicenter prospective study in Korea. J Clin Gastroenterol 2014;48:e12–8. 23632355 10.1097/MCG.0b013e31828f4bc9

[5] Lacy BE Weiser KT Kennedy AT Functional dyspepsia: the economic impact to patients. Aliment Pharmacol Ther 2013;38:170–7. 23725230 10.1111/apt.12355

[6] Sha W Pasricha PJ Chen JD Correlations among electrogastrogram, gastric dysmotility, and duodenal dysmotility in patients with functional dyspepsia. J Clin Gastroenterol 2009;43:716–22. 19247205 10.1097/MCG.0b013e31818b8ed9

[7] Vanheel H Carbone F Valvekens L Pathophysiological abnormalities in functional dyspepsia subgroups according to the Rome III criteria. Am J Gastroenterol 2017;112:132–40. 27958284 10.1038/ajg.2016.499

[8] Schmulson MJ Drossman DA What is new in Rome IV. J Neurogastroenterol Motil 2017;23:151–63. 28274109 10.5056/jnm16214 PMC5383110

[9] Matsuzaki J Suzuki H Asakura K Classification of functional dyspepsia based on concomitant bowel symptoms. Neurogastroenterol Motil 2012;24: 325-e164. 10.1111/j.1365-2982.2011.01859.x PMC3386482 22235936

[10] Sarnelli G Caenepeel P Geypens B Symptoms associated with impaired gastric emptying of solids and liquids in functional dyspepsia. Am J Gastroenterol 2003;98:783–8. 12738456 10.1111/j.1572-0241.2003.07389.x

[11] Mazzoleni LE Sander GB Francesconi CF Helicobacter pylori eradication in functional dyspepsia: HEROES trial. Arch Intern Med 2011;171:1929–36. 22123802 10.1001/archinternmed.2011.533

[12] Wang WH Huang JQ Zheng GF Effects of proton-pump inhibitors on functional dyspepsia: a meta-analysis of randomized placebo-controlled trials. Clin Gastroenterol Hepatol 2007;5:178–85. 17174612 10.1016/j.cgh.2006.09.012

[13] Talley NJ Locke GR Saito YA Effect of Amitriptyline and Escitalopram on functional dyspepsia: a multicenter, randomized controlled study. Gastroenterology 2015;149:340–9. 25921377 10.1053/j.gastro.2015.04.020 PMC4516571

[14] Matsueda K Hongo M Tack J A placebo-controlled trial of acotiamide for meal-related symptoms of functional dyspepsia. Gut 2012;61:821–8. 22157329 10.1136/gutjnl-2011-301454 PMC3345932

[15] Gan Y Liu H Yang L Effect of banxiaxiexin tang on treatment of functional dyspepsia: a meta-analysis of randomized controlled trials. J Tradit Chin Med 2014;34:140–4. 24783922 10.1016/s0254-6272(14)60067-4

[16] Chu MHK Wu IXY Ho RST Chinese herbal medicine for functional dyspepsia: systematic review of systematic reviews. Therap Adv Gastroenterol 2018;11: doi:10.1177/1756284818785573. 10.1177/1756284818785573 PMC6048609 30034530

[17] Oikawa T Ito G Hoshino T Hangekobokuto; (Banxia-houpo-tang), a Kampo medicine that treats functional dyspepsia. Evid Based Complement Alternat Med 2009;6:375–8. 18955239 10.1093/ecam/nem101 PMC2722198

[18] Yoon JH Kim JS Ryu BH An experimental study on the component variation of Naesowhajung-tang by the three types of extraction method and the effects of each type on the gastrointestinal tract. J Int Korean Med 2001;22:29–38.

[19] Kim JY Lee JY Yoon SH Effect of Naesowhajung-tang on Electrogastrography in children with functional dyspepsia. J Korean Orient Pediatr 2002;16:199–213.

[20] Zhang SS Zhao LQ Wang HB Efficacy of Gastrosis No.1 compound on functional dyspepsia of spleen and stomach deficiency-cold syndrome: a multi-center, double-blind, placebo-controlled clinical trial. Chin J Integr Med 2013;19:498–504. 23818201 10.1007/s11655-013-1503-x

[21] Hwang DY Bangyakhappyeon. Seoul: Younglimsa; 2002.

[22] Zhang S Zhao L Wang H Efficacy of modified LiuJunZi decoction on functional dyspepsia of spleen-deficiency and qi-stagnation syndrome: a randomized controlled trial. BMC Complement Altern Med 2013;13:54. 23453018 10.1186/1472-6882-13-54 PMC3599864

[23] Veldhuyzen van Zanten SJ Chiba N Armstrong D Validation of a 7-point Global Overall Symptom scale to measure the severity of dyspepsia symptoms in clinical trials. Aliment Pharmacol Ther 2006;23:521–9. 16441473 10.1111/j.1365-2036.2006.02774.x

[24] Zhu WF Gao EX Ji SL Textbook of Traditional Chinese Medical Diagnosis: Etiological Factors and Qi-Blood-Fluid Pattern Identifying Method. Beijing: Ren min wei sheng chu ban she; 2002 569–655.

[25] Park YJ Lim JS Park YB Development of a valid and reliable food retention questionnaire. Eur J Integr Med 2013;5:432–7.

[26] Park YJ Park JS Kim MY Development of a valid and reliable phlegm pattern questionnaire. J Altern Complement Med 2011;17:851–8. 21834661 10.1089/acm.2010.0504

[27] Park JS Yang DH Y. KM Development of questionnaire for Damum patternization. J Korea Instit Orient Med Diagn 2006;10:64–77.

[28] Lee EH Hahm KB Lee JH Development and validation of a functional dyspepsia-related quality of life (FD-QOL) scale in South Korea. J Gastroenterol Hepatol 2006;21:268–74. 16460485 10.1111/j.1440-1746.2006.04196.x

[29] Weimer K Horing B Muth ER How to study placebo responses in motion sickness with a rotation chair paradigm in healthy participants. J Vis Exp 2014;doi:10.3791/52471. 10.3791/52471 PMC4396966 25549015

[30] Weimer K Sauer H Horing B Impaired gastric myoelectrical reactivity in children and adolescents with obesity compared to normal-weight controls. Nutrients 2018;doi:10.3390/nu10060699. 10.3390/nu10060699 PMC6024785 29857470

[31] Lee H Jung HK Huh KC Current status of functional dyspepsia in Korea. Korean J Intern Med 2014;29:156–65. 24648796 10.3904/kjim.2014.29.2.156 PMC3956983

[32] Brook RA Kleinman NL Choung RS Functional dyspepsia impacts absenteeism and direct and indirect costs. Clin Gastroenterol Hepatol 2010;8:498–503. 20304102 10.1016/j.cgh.2010.03.003

[33] Jee SR Jung HK Min BH Guidelines for the treatment of functional dyspepsia. Korean J Gastroenterol 2011;57:67–81. 21350319 10.4166/kjg.2011.57.2.67

[34] Chiarioni G Pesce M Fantin A Complementary and alternative treatment in functional dyspepsia. United European Gastroenterol J 2018;6:5–12. 10.1177/2050640617724061 PMC5802680 29435308

[35] Guo Y Zhu J Su X Efficacy of Chinese herbal medicine in functional dyspepsia: a meta-analysis of randomized, double-blind, placebo-controlled trials. J Tradit Chin Med Sci 2016;3:147–56.

[36] Kim JS Yoon SH Effect of Naesosan on gastric motlity between normal inatact and antral dilatated rats. Korean J Orient Int Med 2008;29:117–29.

[37] Yang SM Ryu BH Park DW A experimental study on the effects of DAEWHAJUNGEUM. J Korean Med 1997;18:82–96.

[38] Gagnier JJ Boon H Rochon P Recommendations for reporting randomized controlled trials of herbal interventions: explanation and elaboration. J Clin Epidemiol 2006;59:1134–49. 17027423 10.1016/j.jclinepi.2005.12.020

[39] Schulz KF Altman DG Moher D CONSORT 2010 Statement: updated guidelines for reporting parallel group randomised trials. BMJ 2010;340:c332. 20332509 10.1136/bmj.c332 PMC2844940

[40] Chang FY Electrogastrography: basic knowledge, recording, processing and its clinical applications. J Gastroenterol Hepatol 2005;20:502–16. 15836697 10.1111/j.1440-1746.2004.03751.x

[41] Yin J Chen JD Roles of interstitial cells of Cajal in regulating gastrointestinal motility: in vitro versus in vivo studies. J Cell Mol Med 2008;12:1118–29. 18429936 10.1111/j.1582-4934.2008.00352.x PMC3865654

[42] Friesen CA Lin Z Hyman PE Electrogastrography in pediatric functional dyspepsia: relationship to gastric emptying and symptom severity. J Pediatr Gastroenterol Nutr 2006;42:265–9. 16540794 10.1097/01.mpg.0000189367.99416.5e

[43] Park JW Ko SJ Han G The effects of Banha-sasim-tang on dyspeptic symptoms and gastric motility in cases of functional dyspepsia: a randomized, double-blind, placebo-controlled, and two-center trial. Evid Based Complement Alternat Med 2013;doi:10.1155/2013/265035. 10.1155/2013/265035 PMC3686053 23861702

[44] Lee HH Jung HK Choi MG Guideline recommendation for endpoints used in clinical trials for functional dyspepsia. Korean J Gastroenterol 2018;72:170–8. 30419642 10.4166/kjg.2018.72.4.170

[45] Xiao Y Liu YY Yu KQ Chinese herbal medicine liu jun zi tang and xiang sha liu jun zi tang for functional dyspepsia: meta-analysis of randomized controlled trials. Evid Based Complement Alternat Med 2012;2012: doi: 10.1155/2012/936459. 10.1155/2012/936459 PMC3530827 23304226

